# Supplementary material for: Regulation of MT1-MMP Activity through Its Association with ERMs
Source: Cells. 2020 Feb 3;9(2):348. doi: 10.3390/cells9020348 (PMC7072721; doi:10.3390/cells9020348)
Supplement: Supplementary file 1 [file cells-09-00348-s001.pdf]

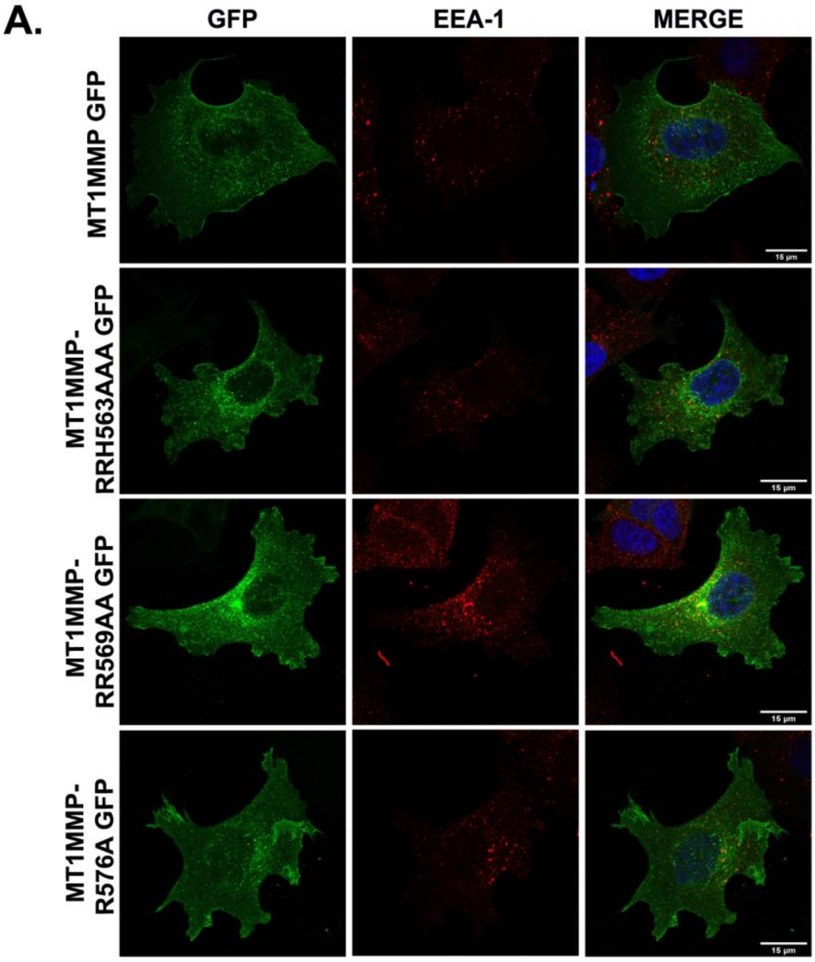
A.
GFP
EEA-1
MERGE
MT1MMP GFP
MT1MMP-RRH563AAA GFP
MT1MMP-RR569AA GFP
MT1MMP-R576A GFP
15 µm
15 µm
15 µm
15 µm

B.

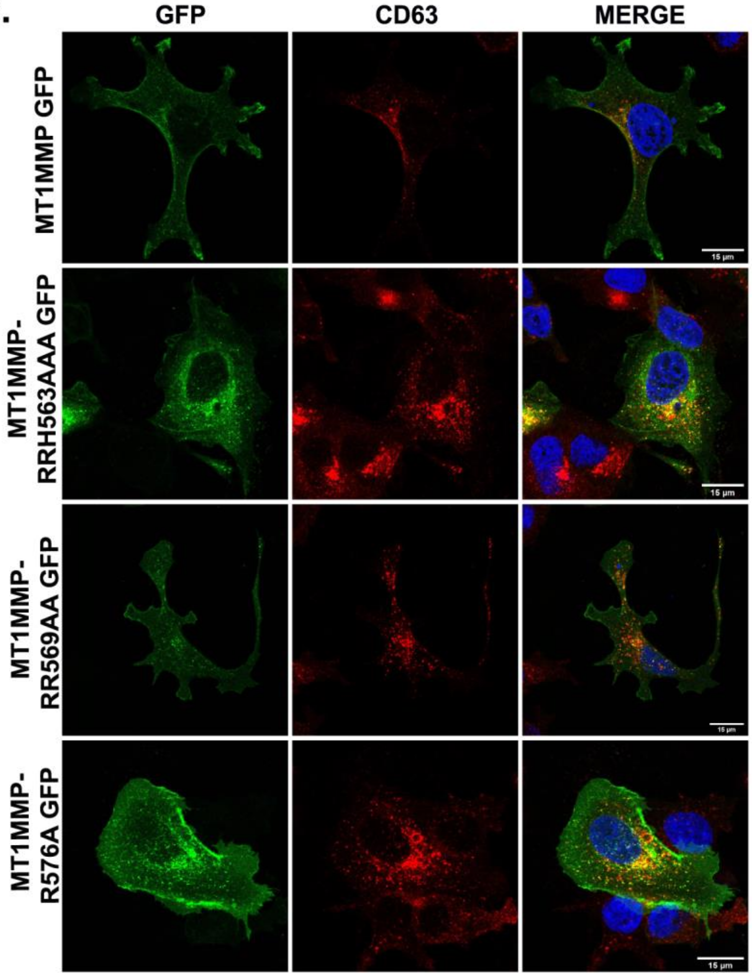
GFP
CD63
MERGE
MT1MMP GFP
MT1MMP-RRH563AAA GFP
MT1MMP-RR569AA GFP
MT1MMP-R576A GFP
15 µm

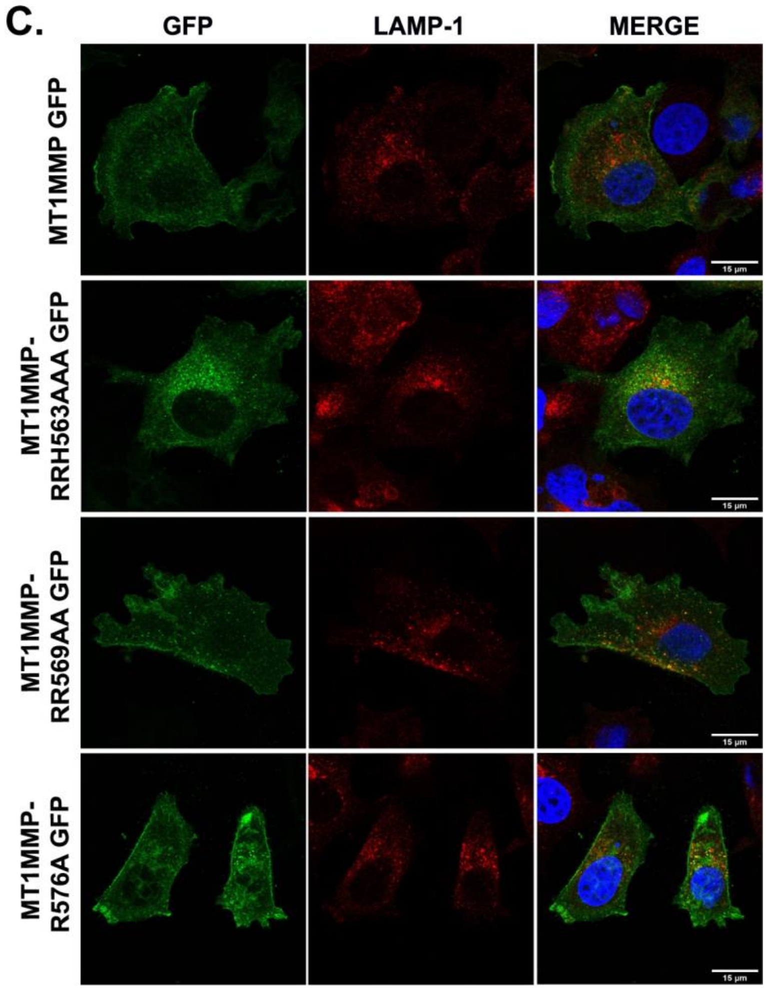

Figure S1. MCF-7 cells were transfected with mEGFP-tagged MT1-MMP constructs and plated onto 20 μg/mL collagen I-coated coverslips, fixed and labeled with anti-EEA1 (**A**), anti-CD63 (**B**) or anti-LAMP-1 (**C**) in red. Bars 15 μm.

A.

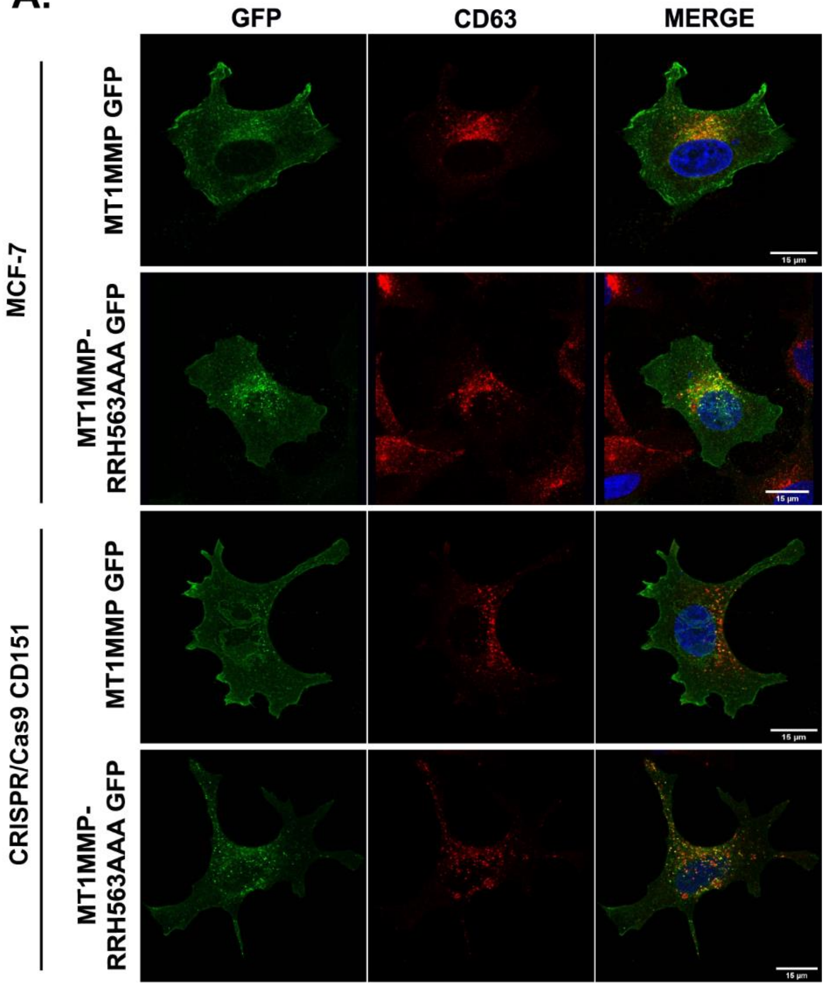
GFP
CD63
MERGE
MCF-7
MT1MMP GFP
MT1MMP-RRH563AAA GFP
CRISPR/Cas9 CD151
MT1MMP GFP
MT1MMP-RRH563AAA GFP
15 µm

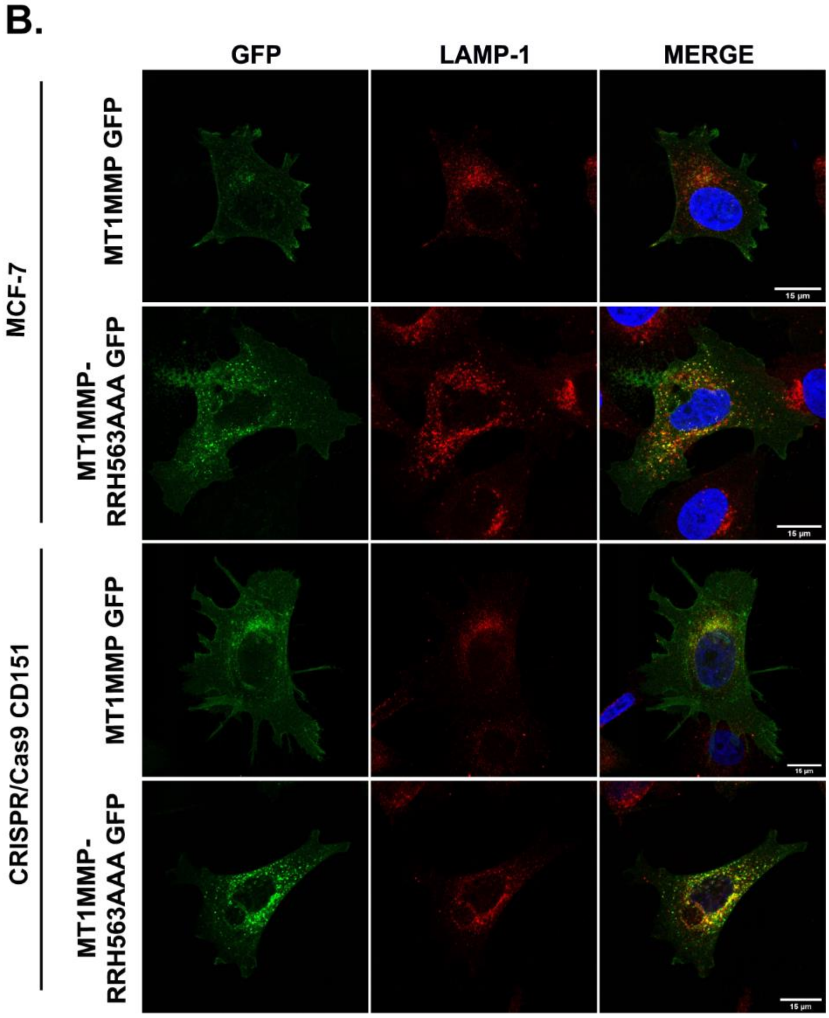

Figure S2. MCF-7 wt or CRISPR/Cas9 CD151 cells were transfected with mEGFP-tagged MT1-MMP constructs (wt or RRH563AAA) and plated onto 20µg/ml collagen I-coated coverslips, fixed and labeled with anti-CD63 (**A**) or anti-LAMP-1 (**B**) in red. Bars 15 µm.
